# Aortic Valve Predilatation with a Small Balloon, without Rapid Pacing, prior to Transfemoral Transcatheter Aortic Valve Replacement

**DOI:** 10.1155/2018/1080597

**Published:** 2018-04-30

**Authors:** Anupama Shivaraju, Christian Thilo, Neal Sawlani, Ilka Ott, Heribert Schunkert, Wolfgang von Scheidt, Adnan Kastrati, Albert Markus Kasel

**Affiliations:** ^1^Deutsches Herzzentrum München, Department for Cardiovascular Diseases, Technische Universität München, Lazarettstr. 36, 80636 Munich, Germany; ^2^Department of Cardiology, Advocate Christ Medical Center, 4440 W. 95th Street, Oak Lawn, IL, USA; ^3^Department of Cardiology, Klinikum Augsburg, Herzzentrum Augsburg-Schwaben, Stenglinstr. 2, 86156 Augsburg, Germany; ^4^Deutsches Zentrum für Herz- und Kreislaufforschung (DZHK), Munich Heart Alliance, Munich, Germany

## Abstract

**Objectives:**

The aim of this study is to assess the feasibility and clinical outcome of transcatheter aortic valve replacement (TAVR) using aortic valve predilatation (AVPD) with a small, nonocclusive balloon.

**Background:**

Balloon aortic valvuloplasty (BAV) under rapid pacing is generally performed in TAVR to ensure the passage and sufficient deployment of the prosthesis in the stenotic AV. BAV may cause serious complications, such as left ventricular stunning or cerebrovascular embolism.

**Methods:**

A cohort of 50 consecutive patients with severe aortic stenosis underwent transfemoral TAVR with the Edwards Sapien 3-heart valve. All patients underwent AVPD with a small, nonocclusive balloon (12 × 60 or 14 × 60 mm) without rapid pacing. Procedural data and clinical outcomes were analyzed.

**Results:**

The mean age of the cohort was 81 ± 6 years and the mean logistic EuroSCORE (European System for Cardiac Operative Risk Evaluation) was 13 ± 9. Crossing the AV and prosthesis implantation was successful in all cases. The postprocedural mean AV gradient was 12 ± 5 mmHg. There were no cases of aortic regurgitation ≥ grade 2. No periprocedural stroke occurred. One patient (2%) with chronic atrial fibrillation displayed a transient Wernicke aphasia occurring more than 24 hours after TAVR. Mortality was 0% at 30 days after procedure.

**Conclusion:**

In TAVR, AVPD with a small, nonocclusive balloon can be safely performed. By avoiding rapid pacing, this technique may be a valid alternative to traditional BAV. Whether or not the use of APVD without rapid pacing translates into less periprocedural complications needs to be assessed in future studies.

## 1. Introduction

Transfemoral transcatheter aortic valve replacement (TAVR) has evolved into the standard of care for patients with severe, symptomatic aortic stenosis (AS) at intermediate, inoperable, or high surgical risk [1]. As device caliber and technological improvements continue to garner increasing attention, some cornerstones of transfemoral implantation techniques remain unchanged. Such principles are rapid pacing and balloon aortic valvuloplasty (BAV) prior to valve implantation [2, 3]. However, the impact of transient ventricular stunning during rapid pacing is unclear, and BAV during TAVR may contribute to cerebral microembolization of calcified debris from the aortic valve [4, 5]. The aim of our current study is to assess the safety and feasibility of AVPD prior to TAVR using a small and nonocclusive balloon without the use of rapid ventricular pacing.

## 2. Methods

### 2.1. Patient Selection and Preparation

From February 2014 until June 2014, we prospectively evaluated and treated 50 consecutive patients with severe, symptomatic AS using AVPD prior to implantation of the Sapien 3 (Edwards Lifesciences, Irvine, CA, USA) transcatheter heart valve (THV).

All patients undergoing TAVR were evaluated with a transthoracic echo (TTE), coronary angiogram, and an ECG gated 384-slice, multidetector computed tomography angiogram (CTA) of the heart and thoracic, abdominal and bilateral lower extremity vasculature. At the time of the procedure, an aortic root and selective iliofemoral angiograms were performed. The procedure was performed under conscious sedation in majority of the cases. A TTE was obtained on all patients on postprocedure day one to assess the aortic valve anatomy and function including obtaining hemodynamic measurements.

### 2.2. TAVR Procedures

**Figure 1:**
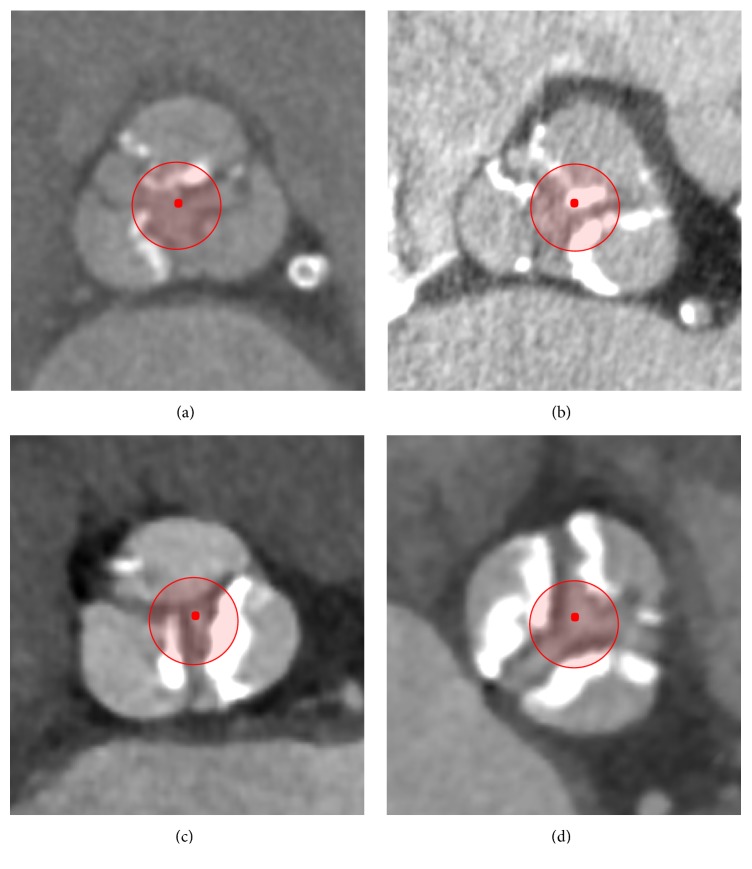
CT-imaging of aortic valves with different degree of calcification. Schematic effect of AVPD with a small, “nonocclusive” balloon.

**Figure 2:**
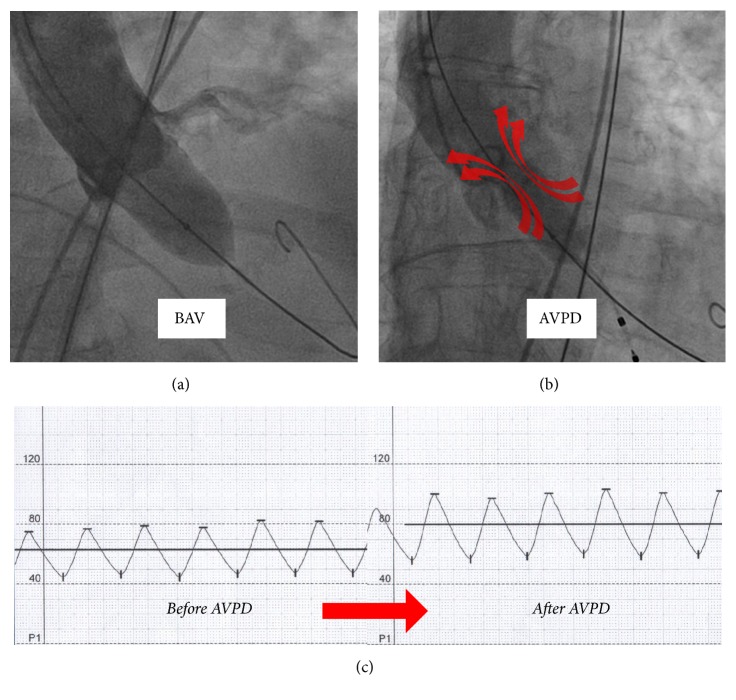
(a) BAV with a normal “occlusive” valvuloplasty-balloon. (b) AVPD with a small, “nonocclusive”-balloon. (c) Hemodynamic effects of AVPD.

After crossing the AV, traditionally BAV is performed using an occlusive balloon (20, 23, or 25 × 40 mm) under rapid ventricular pacing. In our study, however, a nonocclusive balloon (12 × 60 or 14 × 60 mm; Osypka AG, Rheinfelden-Herten, Germany) was used for AVPD (Figure 1). No rapid ventricular pacing was performed during AVPD. A comparison of balloon sizes in relation to aortic valve anatomy is provided in Figures 2(a) and 2(b). An example of pre- and post-AVPD hemodynamic data is shown in Figure 2(c). Thereafter, the Sapien 3 valve was inserted and positioned across the AV annulus under fluoroscopy guidance and deployed under rapid ventricular pacing.

### 2.3. Procedural Endpoints and Definitions

The primary endpoint was device success. The secondary endpoints were disabling stroke, nondisabling stroke, transient ischemic attack (TIA), and procedural mortality. Device success, disabling stroke, nondisabling stroke, TIA, and procedural mortality were all defined using the VARC2 definitions [6]. Device success was defined as absence of procedural mortality, correct positioning of a single prosthetic heart valve, and intended performance of the implanted prosthetic valve (no patient-prosthesis mismatch, mean AV gradient < 20 mmHg, or peak velocity < 3 m/s and no moderate or severe prosthetic valve regurgitation) [6]. Procedural mortality was defined as all-cause mortality within 30 days or during the index procedure hospitalization [6].

### 2.4. Statistical Analysis

Continuous data are presented as mean ± standard deviation, and categorical data are presented as a number and/or percentages. All analysis was performed using XLSTAT for Microsoft Excel 2010 (Microsoft Corporation, Seattle, WA, USA).

## 3. Results

### 3.1. Baseline Characteristics

The baseline characteristics of the study population are shown in Table 1. The mean patient age was 81 ± 6. Half our patients were women. The mean logistic EuroSCORE (European System for Cardiac Operative Risk Evaluation) was 13 ± 9. The mean AV gradient was 39.9 ± 15.7 mmHg and aortic valve area 0.8 ± 0.2 cm^2^, indicating severe aortic stenosis.

### 3.2. Procedural Data

AVPD was performed using a 12 × 60 mm or 14 × 60 mm balloon in 96% of the cases, and in 2 cases (4%) a 12 × 40 mm balloon was used. Procedural data is depicted in Table 2. The mean contrast volume and fluoroscopy time were 108 ± 35 mL and 14 ± 5 minutes, respectively; mean procedural duration was 60 ± 18 minutes.

### 3.3. Procedural Outcomes

Device success was achieved in all cases (100%). No periprocedural stroke, disabling stroke, or TIA occurred. One case (2%) of nondisabling stroke (transient Wernicke aphasia) occurred more than 24 hours after TAVR in a patient with chronic atrial fibrillation. Procedural and 30-day mortality was 0% (Table 3). The patients' hemodynamics was stable during the procedure in all cases without the need for additional inotropic medications during AVPD or THV implantation. The mean AV gradient was reduced from 39.9 ± 15.7 mmHg to 12.3 ± 4.7 mmHg after THV implantation; these gradients were measured on pre- and postprocedure echocardiography. No case of aortic regurgitation grade ≥ 2 occurred (Table 3).

## 4. Discussion

TAVR as a treatment choice for severe AS is an excellent option for patients with inoperable or high surgical risk, but periprocedural stroke remains a concern compared to surgical aortic valve replacement [1]. BAV is usually performed to ensure the passage of the THV through the stenotic aortic valve. Another aim of BAV is to improve the compliance of the rigid, calcified AV leaflets and to increase the aortic valve area [7, 8]. With BAV, a sufficient prosthesis deployment is assured, which is of particular importance for self-expanding THV exerting less radial force. Finally, BAV may be used as an additional sizing modality for prosthesis selection in cases with borderline or questionable annulus size. The mechanisms underlying successful BAV are mainly commissural separation and perhaps more importantly intraleaflet fracturing of calcific nodules [8–10]. Microembolization of debris is thought to occur during multiple phases of the procedure, such as crossing the aortic valve and ascending aorta with different wires, performing BAV, and positioning of the THV [4, 11]. These microembolizations may account to strokes associated with TAVR. Multiple studies have identified silent diffuse embolic strokes on magnetic resonance imaging studies [5]. Moreover, prior studies have demonstrated the advantages of avoiding BAV in reducing stroke [12, 13]. Reports have also been published on deleterious outcomes with BAV prior to TAVR [14]. A current meta-analysis suggested a 30-day stroke rate between 3.2% and 4.2% using the Edwards Sapien valve [15]. A recently published multicenter trial using the Edwards Sapien 3 valve observed a 30-day mortality of 2.1% and a rate of disabling strokes of 0% [16]. The nondisabling stroke rate was 1% in their transfemoral cohort [16].

One drawback of BAV is the need for rapid ventricular pacing to allow hemodynamic arrest of the heart. This ensures optimal balloon positioning and avoids excessive movements of the balloon during inflation. These balloon movements may also contribute to embolization of calcified material from the stenotic valve [8]. However, rapid left ventricular pacing carries several risks. First, it may induce ventricular fibrillation in rare cases [17]. Second, it purposely decreases cardiac output temporarily with the potential of hemodynamic instability, especially in patients with aortic stenosis and hypertrophied left ventricle [18]. Previous studies have also shown that rapid pacing is associated with smaller post-BAV AV area compared to BAV alone [7, 13]. One explanation of this phenomenon may be that rapid ventricular pacing provokes temporary myocardial stunning, thereby decreasing contractility and flow, resulting in the calculation of a smaller aortic valve area [18]. In patients with severely reduced LV function, any additional rapid pacing may be harmful due to this myocardial stunning. These patients might benefit from performing AVPD without rapid ventricular pacing instead of traditional BAV.

In recent reports, operators perform direct TAVR without BAV, also avoiding rapid ventricular pacing [13]. No increase in adverse outcomes was seen with direct valve implantation of the Edwards Sapien XT valve [13]. It should be noted, however, that these were patients with low to moderate calcification of the AV. Furthermore, it has to be considered that most of the TAVR valves cannot be withdrawn once they have entered the body. Thus, failure of crossing the aortic valve without predilatation makes the procedure much more complex. Our prospective analysis shows excellent procedural success and device success rates using AVPD without rapid pacing (Table 2). Passage of the THV through the stenotic AV was achieved on the first attempt in all patients, even in cases with severely calcified valves. In each case, single balloon dilation was employed and the rate of clinical stroke was very low. During the 30 day period, we observed no disabling stroke, and only one nondisabling stroke that happened more than 24 h after TAVR in a patient with chronic atrial fibrillation. Despite the small study population, this allows us to hypothesize that performing AVPD with a small balloon is less aggressive in comparison to BAV and may reduce mortality as well as both the degree of microembolization and clinical stroke. However, larger cohorts and corroborative imaging will be needed for a more definitive conclusion about the impact of AVPD on stroke and mortality.

## 5. Study Limitations

This prospective study reflects a single-center experience using AVPD in a limited number of patients without a control group. Since this is not a randomized study, selection bias is inherent and may have influenced our findings.

## 6. Conclusion

The use of small balloons for AVPD prior to positioning and implantation of a transfemoral THV is a safe and feasible technique. It eliminates the need for rapid pacing without compromising procedural outcomes. Improvement in the TAVR procedure with refinement of the device is promising, but the advancements in procedural techniques described here are also necessary to improve patient outcomes. Future randomized trials are required to determine the impact of AVPD without rapid pacing in comparison to the traditional BAV and direct THV implantation on hemodynamic and clinical outcomes, especially on silent and clinical cerebral ischemia.

## Figures and Tables

**Table 1:** Demographic and clinical characteristics of the study population.

Baseline characteristics	
	Overall population (*N* = 50)
Age, yrs	80.8 ± 5.9
Female, *n* (%)	25 (50)
BMI, kg/m2	27.3 ± 5.9
Diabetes, *n* (%)	18 (32)
Dyslipidemia, *n* (%)	32 (64)
Hypertension, *n* (%)	45 (90)
Tobacco use, *n* (%)	11 (22)
Coronary artery disease, *n* (%)	35 (70)
Prior MI, *n* (%)	3 (6)
Prior CABG, *n* (%)	3 (6)
Prior PCI, *n* (%)	19 (38)
Prior stroke or TIA, *n* (%)	4 (8)
Peripheral vascular disease, *n* (%)	7 (14)
COPD, *n* (%)	7 (14)
Baseline creatinine, mg/dL	1.1 ± 0.3
Logistic EuroSCORE 2	5.6 ± 5.0
EuroSCORE	13.1 ± 8.8
LVEF, %	52.2 ± 12.5
Maximum aortic gradient, mmHg	65.2 ± 24.0
Mean aortic gradient, mmHg	39.9 ± 15.7
Aortic valve area, cm^2^	0.8 ± 0.2

Values are *n* (%) or mean ± standard deviation. BMI = body mass index, MI = myocardial infarction; CABG = coronary artery bypass graft, PCI = percutaneous coronary intervention, TIA = transient ischemic attack, COPD = chronic obstructive pulmonary disease, and LVEF = left ventricular ejection fraction.

**Table 2:** Procedural data.

Procedural data	
	Overall population (*N* = 50)
Contrast volume, mL	107.9 ± 35.3
Radiation dose, Gy	32402.5 ± 26989.7
X-ray time, min	13.9 ± 5.2
Length of procedure, min	59.9 ± 17.8
Balloon Size, *n* (%)	
12 × 40 mm	2 (4)
12 × 60 mm	32 (64)
14 × 60 mm	16 (32)
Valve Size, *n* (%)	
SAPIEN 3 23 mm	22 (44)
SAPIEN 3 26 mm	19 (38)
SAPIEN 3 29 mm	9 (18)

Values are *n* (%) or mean ± SD. F = French.

**Table 3:** Procedural outcomes.

Procedural outcomes	
Complication, *n* (%)	Overall population (*N* = 50)
Device success	50 (100)
Aortic regurgitation ≥ grade 2	0 (0)
Mean AV gradient, mmHg	12.3 ± 4.7
Myocardial infarction	1 (2)
Periprocedural stroke	0 (0)
Disabling stroke	0 (0)
Nondisabling stroke	1 (2)
TIA	0 (0)
BARC life threatening/disabling bleeding	0 (0)
BARC major bleeding	4 (8)
BARC minor bleeding	5 (10)
VARC2 major vascular complication	0 (0)
VARC2 minor vascular complication	7 (14)
Permanent pacemaker implant	8 (16)
Acute kidney injury (RIFLE Classification)	5 (10)
*Stage 1 (risk)*	2 (4)
*Stage 2 (injury)*	3 (6)
*Stage 3 (failure)*	0 (0)
Procedural mortality	0 (0)

Values are *n* (%). AV = aortic valve, TIA = transient ischemic attack, BARC = bleeding academic research consortium, VARC = valve academic research consortium, RIFLE = risk, injury, failure, loss of kidney function, and end-stage kidney disease.
